# Psychometric properties of an English short version of the Trier Inventory for chronic Stress

**DOI:** 10.1186/s12874-020-01156-y

**Published:** 2020-12-16

**Authors:** K. Petrowski, E. Braehler, B. Schmalbach, A. Hinz, C. Bastianon, T. Ritz

**Affiliations:** 1 Medical Psychology and Medical Sociology, University Medical Center of the Johannes Gutenberg University of Mainz, Mainz, Germany; 2 Department of Internal Medicine III, Carl Gustav Carus University Hospital, Fetscherstrasse 74, 01307 Dresden, Germany; 3 Department of Psychosomatic Medicine and Psychotherapy, University Medical Center of the Johannes Gutenberg University of Mainz, Mainz, Germany; 4 Department of Medical Psychology and Medical Sociology, University of Leipzig, Leipzig, Germany; 5 Department of Psychology, Southern Methodist University, Dallas, TX USA

**Keywords:** Chronic stress, Screening questionnaire, Factor analysis, Measurement invariance, Trier inventory for chronic stress

## Abstract

**Background:**

Although a variety of instruments are available that capture stress experience, the assessment of chronic stress has been hindered by the lack of economical screening instruments. Recently, an English-language version of the Trier Inventory for Chronic Stress (TICS-EN) consisting of 57 items according to a systemic-requirement-resource model of health in nine subdomains of the chronic stress experience has been introduced.

**Methods:**

We constructed a new 9-item short version of the TICS covering all nine subdomains and evaluated it in two samples (total *N* = 685). We then used confirmatory factor analysis to check factorial validity.

**Results:**

This version showed a highly satisfactory model fit, was invariant across participant gender, demonstrated a very high correlation with the original TICS (*r* = .94), and showed a moderate correlation (*r* = .58) with a measure of perceived stress in the past month.

**Conclusions:**

Therefore, this theoretically driven instrument can be recommended as a short version of the TICS in English language.

## Background

According to the Federal Institute for Occupational Safety and Health, the levels of perceived stress have significantly increased since the early 2000s [1]. Consequences of long-lasting chronic stress constitute an increased risk for impaired psychological wellbeing and acute physical illnesses [2, 3]. Associations between psychosocial stress and depression, cardiovascular disease, sleep disorders or chronic pain are well-established [4–9]. Elevated stress levels are also a factor in susceptibility to upper respiratory tract infections, asthma, herpes viral infections, autoimmune diseases, and delayed wound-healing [9]. Accordingly, measuring chronic stress with a brief and efficient instrument is valuable to multiple disciplines [10].

An in-depth overview of general- and area-specific stress instruments is provided by Cohen and colleagues [3]. However, the focus on acute stress has often overlooked chronic, long-term stress. For chronic stress, the Perceived Stress Questionnaire [11, 12] provides a general retrospective one-year evaluation in addition to the last four, six, and 8 weeks without area specificity. The Trier Inventory for Chronic Stress (TICS) [13] is an instrument that targets explicitly area-specific chronic stress including Work Overload, Social Overload, Pressure to Perform, Work Discontent, Excessive Demands from Work, Lack of Social Recognition, Social Tensions, Social Isolation, and Chronic Worrying. These nine domains of chronic stress are measured by 57 items [13, 14], which were selected in accordance with the systemic-requirement-resource model of health [15]. Schulz, Schlotz, and Becker [13] developed the TICS scales based on this model and thus assumed high content validity as a rational response. In a representative German study the confirmatory factor analysis (CFA) provided evidence for a good factorial validity [14].

A 12-item version of the TICS (Short Screening Scale for Chronic Stress, SSCS) was developed by the original authors [13] to meet the needs for a brief chronic stress instrument for applied research and practitioners. Items were selected based on factor loadings of the strong, unrotated first factor (explained variance 28.4%). However, this empirical item selection included only five of the original nine stress domains from the full 57-item scale in the SSCS. With the stress domains represented in the SSCS (Chronic Worry, Work Overload, Excessive Demands from Work, and Lack of Social Recognition) the SSCS correlated moderately to highly with the original nine subscales of the 57-item (*r* = .68–.87). Social Overload retained one item for the SSCS but correlated low with the subscale of the long version of the TICS (*r* = .45) [13]. The four scales not represented in the SSCS, Pressure to Perform, Work Discontent, Social Tensions, and Social Isolation, showed low correlations as well with the 12-item SSCS (*r* = .40–.56) [13]. Therefore, four of the nine theoretically proposed areas of chronic stress are underrepresented in the SSCS. Due to the item selection procedure, the content domain was reduced and thus the validity of the SSCS was weakened [16]. The strength and uniqueness of the original 57-item TICS lies in its area-specific chronic stress assessment based on the theoretical model of health, unfortunately this strength is no longer represented in the short 12-item version (TICS-12, SSCS).

In order to represent the theoretically and empirically supported nine areas of chronic stress, a new short version of the TICS was identified. Petrowski et al. [17]. A representative German sample of *N* = 2473 was used to construct and test the new 9-item TICS. Nine items based on the alphamax algorithm were chosen to represent the nine areas of chronic stress from the original TICS (TICS-57). The one-factor-model of TICS-9 provided a good fit for the latent construct and showed good internal consistency (α = .88). As the original 57-item and 9-items TICS were developed and tested in German, we sought to replicate the findings with the English TICS-EN version [18] and evaluate the psychometric properties of an English-language short form TICS-9.

## Method

### Participants and procedure

The data of Sample 1 was collected at two college campuses in the Eastern and Southwestern region of the USA. Participants were undergraduate introductory psychology students who contributed in return for course credit. The final pooled sample consisted of *n* = 501. They received a data protection declaration that is in agreement with the Helsinki Declaration. The study was approved by the institutional review boards of the involved university institutions and all participants provided written informed consent.

Participants of Sample 2 were recruited in Spring 2019 via Amazon’s Mechanical Turk [19], a crowd-sourcing website. MTurk is an international online platform that allows researchers to post tasks or questionnaires that participants complete in return for payment. In the current study, participants signed up via MTurk and were then directed to the online survey to complete the questionnaire. This survey was only available to participants who were located in the USA and their MTurk approval rating greater than 95%. The questionnaire took approximately 5 min to complete and participants were compensated $0.50 for their time. Sample 2 was collected in order to evaluate the factorial structure in a study. The final sample consisted of *N* = 184 participants.

The study was approved by the ethic review boards of Landesärztekammer Rheinland-Pfalz, Germany, and all participants provided informed consent online by agreeing to take part in the study (Table 1).

**Table 1:** Sample description

	Sample 1 (*n* = 501)			Sample 2 (*n* = 184)		
	*n*	%	TICS	*n*	%	TICS
Gender			M (SD)			M (SD)
Female	366	73.1	2.47 (0.72)	96	52.2	2.44 (0.85)
Male	118	23.6	2.54 (0.70)	88	47.8	2.46 (0.89)
Missing	17	3.4	2.05 (0.84)			
Age (in years)	*M* = 19.97, *SD* = 2.84			*M* = 37.86, *SD* = 11.35		
≤ 20	366	73.1	2.50 (0.70)	2	1.1	3.44 (0.31)
21–25	116	23.2	2.42 (0.79)	16	8.7	2.78 (0.79)
≥ 26	19	3.8	2.37 (0.72)	166	90.2	2.41 (0.87)
Ethnicity						
White	391	78.0	2.49 (0.67)	148	80.4	2.35 (0.81)
Black or African American	22	4.4	2.58 (0.69)	16	8.7	2.26 (0.81)
Asian or Pacific Islander	25	5.0	2.28 (0.81)	6	3.3	2.41 (0.87)
Hispanic or Latino	6	1.2	2.74 (0.44)	13	7.1	2.94 (0.98)
Multi-ethnic	6	1.2	3.67 (0.78)	3	1.6	2.70 (0.39)
Other	25	5.0	2.12 (0.97)			
Missing	26	5.2	2.34 (0.91)	2	1.1	2.67 (0.63)

Note. *TICS* Trier Inventory for Chronic Stress

### Measures

The Trier Inventory for Chronic Stress (TICS) is a standardized German questionnaire that has been tested with respect to its factorial structure and psychometric properties, showing good to very good reliability [14]. Internal consistency (Cronbach’s Alpha, α) was good to very good with values ranging from .84 to .91 (mean of α = .87) [13]. Nine interrelated factors of chronic stress are assessed: Work Overload; Social Overload; Pressure to Perform; Work Discontent; Excessive Demands at Work; Lack of Social Recognition; Social Tensions; Social Isolation; Chronic Worrying. The nine factors were derived from 57 items rated on a five-point rating scale (1–5, labeled as: “never”, “rarely”, “sometimes”, “frequently”, “always”). Participants rated the occurrence/frequency of specific situations with a recall period of the previous 3 months. The 12 items with the highest loadings constitute the short version by the original authors [13]. In addition, a new short version of the TICS was developed based on the alphamax algorithm representing the nine areas of chronic stress of the original TICS. The one-factor-model of this new short version provided a good fit for the latent construct and showed good reliability (α = .88) [17]. After the translation state-of-the-art (see Petrowski et al. [18]), the English version of the Trier Inventory of Chronic Stress (TICS-EN) with 57 items was used in the present study [18].

The Perceived Stress Scale (PSS-10) is the most widely used psychological instrument for measuring perceived stress [20]. Respondents report the degree to which situations in one’s life have been unpredictable, uncontrollable and overloaded in the past month on a 5-point scale (0 = never, 1 = almost never, 2 = sometimes, 3 = fairly often, 4 = very often).

### Statistical analyses

We conducted the analyses in R, using the packages *lavaan*, *lordif*, *MBESS*, and *semTools* [21–24]*.* Participants with missing values on any of the TICS-9 items were excluded from the analysis: seven and eleven participants. In addition, we excluded participants who failed the attention checks utilized in Sample 2 (*n* = 28). Very few participants (less than 5% across all items in both samples) chose the highest response option, making the items essentially ordinally scaled. Previous research suggests that conventional maximum likelihood estimation tends to be inaccurate with four or fewer response categories [25, 26]. Therefore, we used the robust diagonally weighted least squares estimation method [27].

To evaluate model fit we considered the following measures and cutoff values [28–30]: The χ^2^-statistic should ideally be non-significant and is calculated by χ^2^ divided by the degrees of freedom of the model, which should < than 2 to indicate good, or < 3 to indicate acceptable, fit. The Comparative Fit Index (CFI) and the Tucker-Lewis Index (TLI), which should be > .95 for good, or > .90 for acceptable fit, and finally, the Root Mean Square Error of Approximation (RMSEA) and the Standardized Root Mean Square Residual (SRMR), which should be lower than .08 to indicate acceptable, or < .05 to indicate good fit. Additionally, we report the 90% confidence interval for the RMSEA. In line with Dunn et al. [31], we tested reliability using McDonald’s ω [32], accompanied by a 95% confidence interval.

In order to test for measurement invariance across gender groups, we used the approach of comparing increasingly constrained models as described by Milfont et al. [33]. Since we were dealing with ordered categorical data we modified the procedure in the way described by Wu et al. [34]: First, we compared the unconstrained (configural) model to a model with item thresholds fixed to be equal across groups. Second, the threshold-invariant model was compared to the metric model (item loadings constrained). Third, the metric model was compared to the scalar model (item intercepts constrained). To evaluate the model comparisons, we primarily used the differences in CFI and gamma hat (GH) between models – which should not exceed .01. Additionally, we tested for significant differences in χ^2^. To avoid selecting a non-invariant marker variable we estimated all factor loadings freely and set the variance of the latent variable to 1 instead. In addition, we analyzed differential item functioning using a logistic ordinal regression framework to be able to pinpoint the origin of whatever instances of measurement non-invariance we encountered [35–37].

## Results

### Item descriptive statistics

**Fig. 1:**
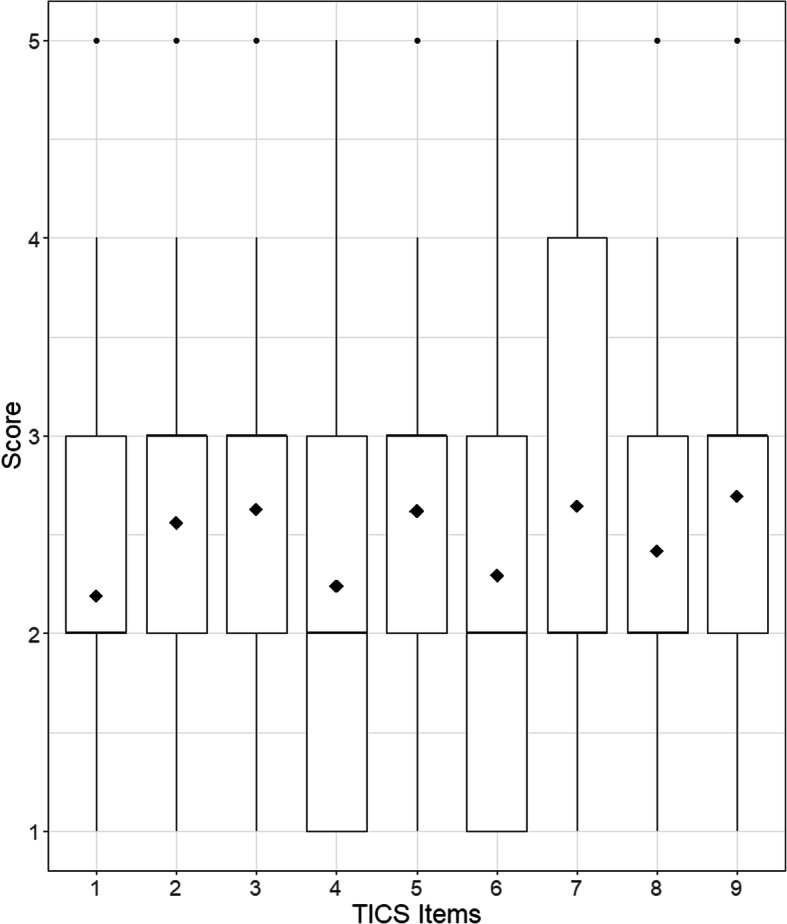
Boxplots of the TICS-9 item distributions. Diamonds represent the item score means

We report descriptive statistics for the TICS-9 items in Table 2 and Fig. 1 and mean scores by sociodemographic group membership in Table 1. All corrected item-total correlations were above the commonly used cutoff value of .50.

**Table 2:** Descriptive statistics for the TICS-9 (Sample 1)

Item	*M*	*SD*	Skewness	Excessive Kurtosis	*r*_*it*_	*CDIF*	*NCDIF*
1	1.838	0.910	0.949	0.467	.537	0.0117	0.0116
2	2.319	1.003	0.414	−0.306	.650	−0.0559	0.0356
3	3.275	1.066	−0.318	−0.358	.543	0.1627	0.1067
4	2.385	1.061	0.475	−0.444	.674	0.0288	0.0066
5	2.218	1.042	0.492	−0.504	.651	0.0082	0.0040
6	2.154	0.993	0.512	−0.510	.608	−0.0222	0.0029
7	2.669	1.184	0.236	−0.787	.594	0.1162	0.0771
8	2.375	1.001	0.406	−0.286	.573	−0.0276	0.0221
9	3.024	1.075	−0.193	−0.508	.580	0.0394	0.0062

Note*. r*_*it*_ Corrected item-total correlation, *CDIF* Compensatory differential item functioning, *NCDIF* Non-compensatory differential item functioning

### CFA and reliability analysis

We report the results of the CFA in Table 3. Model fit was acceptable in both samples: Only the χ^2^-test indicated a significant deviation from the theoretical model. All fit indices presented evidence for acceptable, even good model fit. Internal consistency was satisfactory in both samples: ωSample1 = .868 [.850; .887], ωSample2 = .872 [.816; .927]. In comparison, the 57-item long version, which was included in Sample 1, had a substantially higher reliability coefficient, ω = .969 [.965; .974].

**Table 3:** Model fit in both samples

	χ^2^ (*df*)	*p*	χ^2^/*df*	*CFI*	*TLI*	*RMSEA* [90% *CI*]	*SRMR*
Sample 1	116.878 (27)	< .001	4.329	.988	.984	.082 [.067; .097]	.060
Sample 2	40.614 (27)	.045	1.504	.997	.995	.073 [.011; .117]	.057

### Measurement invariance

Next, we tested for measurement invariance across gender using Sample 1. Since not all items in all groups had sufficient frequencies for all response options, we collapsed the two highest replies “4” and “5”, putting the items on a four-point scale for the analysis of invariance. Utilizing the procedure described above we found evidence for invariance across gender. Only the χ^2^ statistic showed a significant deviation, which was limited to the final comparison. CFI and GH never exceeded the cutoff of .01 between levels of constraints, indicating that women and men do not differ meaningfully in terms of their response behavior to the TICS-9.

**Fig. 2:**
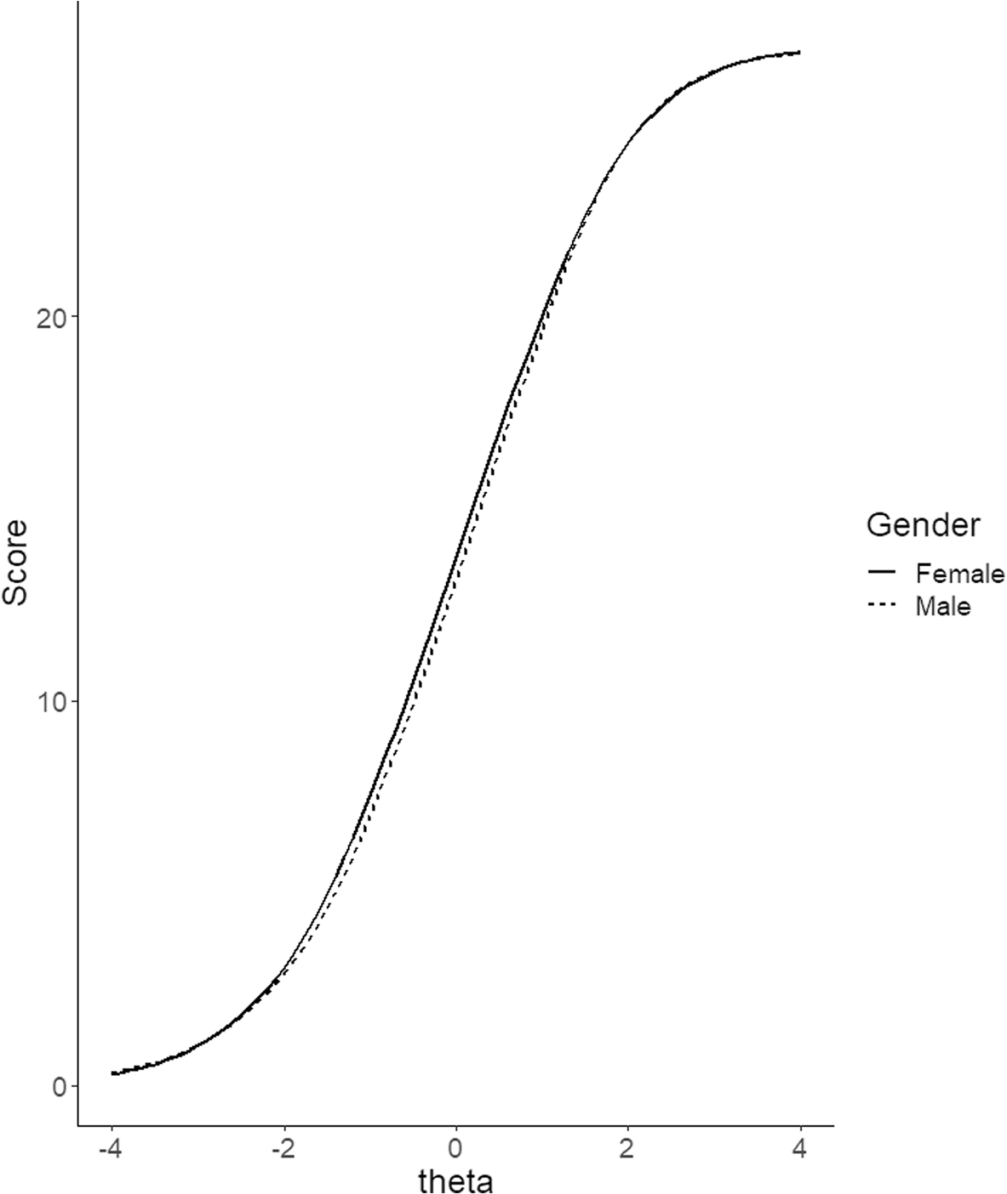
Gender-specific test characteristic curves. The scale range differs from the empirical distribution because items were rescaled to minima of 0 and groups of insufficient size were collapsed

When considering the indices of non-compensatory differential item functioning (NCDIF) presented in Table 2, it becomes clear that most of the gender-specific differences are attributable to Items 3 and 7, with both of them exceeding the cutoff of NCDIF ≤0.054 for four-point items [38]. Thus, item-specific comparisons – specifically for these two items – are discouraged. However, considering the entire scale, differential test functioning (the sum of compensatory differential item functioning (CDIF), which accounts for the different directions of DIF; DTF = 0.2614), was below the critical value of 9 * .054 = 0.486. Thus, overall there was a slight trend for women to choose higher response options than men, given the same trait level of stress (see Fig. 2). However, this difference was so small that it was unlikely to meaningfully influence interpretation of test scores. This was also evident from Fig. 2, which shows response behavior on the scale level across trait values (Table 4).

**Table 4:** Analysis of measurement invariance across gender (Sample 1)

Model	χ^2^ (*df*)	Δχ^2^	Δ*df*	*p*	*CFI*	Δ*CFI*	*GH*	Δ*GH*
Configural inv.	327.328 (56)				.956		.889	
Male	77.251 (28)				.966		.915	
Female	250.077 (28)				.952		.881	
Threshold inv.	340.100 (65)	12.772	9	.173	.955	.001	.888	.001
Loading inv.	346.736 (72)	6.636	7	.468	.955	.000	.888	.000
Intercept inv.	378.449 (80)	31.713	8	< .001	.951	.004	.879	.009

### Validity

In Sample 1, we found a very high correlation between the TICS-9 and the TICS-57, r (499) = .944, *p* < .001. Additionally, in Sample 2, there was a moderately high association of TICS-9 with PSS-10, *r*_Sample2_(182) = .583, *p* < .001.

## Discussion

A reliable nine-item version of the English TICS-EN was constructed for the assessment of chronic stress. The TICS-9-EN captures all areas of the systemic-requirement-resource model of health. Furthermore, the approach used to develop the English version TICS-9 benefited from the recommendations of Smith et al. [39], thus avoiding common methodological sins in developing short form scales [13, 15, 39]. Scales developed using only those items with the highest item-total correlations for a given factor [39–42] may uphold a high internal consistency estimate of reliability, but the content domain is unintentionally constricted and the validity of the short form is diminished [39, 42]. For that reason, the alphamax algorithm was used to maximize reliability while maintaining all nine domains of chronic stress, thus avoiding this common sin. In order to avoid the challenge of impaired content coverage for a construct and inadequate validity from fewer items, TICS-9-EN covered all nine domains of the systemic-requirement-resource model of health [15] with one item each. The strength and uniqueness of the original TICS-57 was based on the theoretical model of health [13, 15] and by maintaining content coverage, this advantage was also conveyed to the TICS-9-EN.

From a practical perspective, our short form combines the validity of the full-length version with an efficient version that is particularly suitable for large multivariable studies. It demonstrations a very good factorial structure and is highly correlated with the 57-item version. Meaningful interpretations can be made based on gender and age differences due to the invariance of the scale. Furthermore, the psychometric properties of the TICS-9-EN are similar to the original German long as well as short version of the TICS [17, 18]: Model fit is good, so is reliability, and the scale can be considered invariant across pertinent sociodemographic groups. Future studies could complement the existing analyses by investigating cultural invariance between the two versions of the instrument.

The study has the strength that separate samples were drawn in order to replicate the factorial structure. However, some limitations need to be acknowledged. While the item selection algorithm maximizes Cronbach’s alpha, at the same time, it increases model fit specifically for a one-factor solution. The improved psychometric properties of the new TICS-9 compared to the SSCS is, consequently, partially a result of statistical methods, see Petrowski et al. [17]. Another limitation is the comparison with the PSS only. Associations with additional instruments for negative affect and chronic stress assessment should be implemented in future studies to fully examine convergent and divergent validity. Future research should demonstrate criterion validity by exploring the relationship. With external ratings of chronic stress. A longitudinal design or a study with repeated measurements would provide opportunities for assessing the factor structures over time, determining retest-reliability, and testing for potential cohort effects.

## Conclusions

In conclusion, our study presents a brief 9-item version of the TICS for measuring chronic stress that is theoretically based and shows strong reliability and emerging evidence for validity. Additionally, it features measurement invariance across participant gender allowing for meaningful interpretations of age and gender differences. Therefore, it can be recommended as an economical screening instrument for multivariable studies in psychology, medicine, and epidemiology.

## Data Availability

Data and materials are available from the corresponding author upon reasonable request.

## Abbreviations

TICS: Trier Inventory for Chronic Stress
CFA: Confirmatory Factor Analysis
PSS: Perceived Stress Scale
CFI: Comparative Fit Index
TLI: Tucker-Lewis Index
RMSEA: Root Mean Squared Error of Approximation
SRMR: Standardized Root Mean Squared Residual
GH: Gamma Hat
CDIF: Compensatory Differential Item Functioning
NCDIF: Non-compensatory Differential Item Functioning
DIF: Differential Item Functioning
DTF: Differential Test Functioning
